# 1902

**Published:** 1903-01-03

**Authors:** 


### Hospital Clinics and Medical Progress.

### 1902.

Our simple duty which is comprised in chronicling from week to week the progress of medical science and the growth of charity, may seem, as indeed it is, a tame and uneventful form of journalism when compared with the more thrilling task of dealing with events which stir the pulses of the world. Yet the year 1902, which will in many ways be marked in history as a famous one, has even in its medical aspects touched upon interests of the supremest importance, interests which have extended far beyond professional circles, while in regard to charity the abundant evidences of this virtue have in certain directions made the past year stand out high above its fellows.

Notwithstanding the fact that the past year has been in every direction one of many expenses—holidays, taxes, ceremonials, entertainments, all of them involving heavy calls upon the pocket—the stream of charity has shown no sign of drying up, the great hospitals which depend upon voluntary contributions having held their ground, while the special funds have prospered exceedingly.

### Hospital Funds.

By dint of energy and enthusiasm, and partly by the stimulating manner of giving adopted by one munificent donor, the old established Hospital Sunday Fund was able to establish a "record," the amount received running up to the surprising total of £62,669, the largest ever collected since the foundation of the Fund. A striking feature of the year in regard to this Fund was the very large increase in the amount collected on Hospital Sunday at the various places of worship. This was without question due in large measure to the energy, eloquence and goodwill of the ministers of every denomination who on that day preached the doctrine of charity; but it can hardly be doubted that the announcement which these preachers were able to make that by the generosity of Mr. George Herring one-fourth would be added to whatever sum might be collected, was a mighty stimulation to good giving. An opportunity of adding 25 per cent. to the value of a gift by the mere act of dropping it into a collecting bag was surely an occasion not lightly to be overlooked. King Edward's Hospital Fund for London has also been successful, far beyond the expectations of many, in its endeavour to free the hospitals from debt. To pay the debts of a number of institutions each of which is under a constant temptation to gather around it fresh liabilities, may to some appear a task of Sisyphus. Yet it is gradually being accomplished, and the fact that last year King Edward's Hospital Fund for London was able to distribute £100,000, shows how great is the vitality of the principles upon which the Fund is founded; those principles of personal service in the cause of the hospitals which have taken active working shape in the foundation of the League of Mercy. The Hospital Saturday Fund, working in its own continuous and systematic fashion, chiefly depending as it does upon weekly collections in workshops and places of business, has also continued its successful and useful career, the sum collected having last year been slightly in excess of that of the year before. Thus does charity prevail.

### Medicine and Surgery.

In medical and surgical work interest has chiefly centred round small-pox, typhoid fever, cancer, tuberculosis, appendicitis, and certain surgical procedures. In regard to none of these can it be said that any striking discovery has been made, yet, in regard to all, there is evidence that progress is taking place in the art of healing.

### Small-pox.

To dwellers in London one of the most marked medical events of the year has been the outbreak of small-pox, which in the early months threatened to become a widespread epidemic. Fortunately, under the double influence of vaccination and isolation, the disease rapidly diminished, and has now, for the present, practically become a thing of the past. The rapid manner, however, in which from small beginnings large areas of the metropolis became seriously permeated with small-pox infection showed how weak is the protection afforded by the sort of vaccination which for some years past has been current among large sections of the population, and how urgently necessary it is that strong measures should be adopted to enforce such a degree of universal and effective vaccination as to rid us of this disgusting and unnecessary pest. It would, we think, be well if those engaged in the collection of statistics on the subject would tell us something authoritative about the social status, the habits of life, and the condition as to settled residence of those who have been affected by this disease. We should be much surprised if it did not turn out that a large and entirely undue proportion of the cases was drawn from that section of the community which may be best described as frequenters of common lodging houses. Much has been done of late years to bring common lodging-houses under better sanitary control. One of the most urgent sanitary wants of the present time is some better control over the frequenters of these houses. We sadly want some systematic arrangement for the identification of tramps. Compared with the mass of the population tramps are but a small fraternity. But they do an amount of harm and cost the community an amount of money, out of all proportion to their number, and still more out of proportion to their value to the community. If only in the name of sanitation their movements ought to be controlled, but in fact they require control on many other grounds. One point of importance to sanitary authorities has been brought out very strongly by the spread of the disease into Essex, for it has been held on good authority that by means of aërial convection the infection of small-pox has been carried across the river from the hospital ships and has been implanted among the dwellers on the Essex shore of the Thames. We can but hope that these assertions may be disproved, for such an exaggerated example of the theory of aërial convection of infection is enough, if generally accepted, to render the position of sanitary authorities, who are bound to provide accommodation for small-pox patients, well-nigh intolerable.

### Enteric Fever.

As the medical history of the late war in South Africa has been gradually unfolded, the question of enteric fever and its prevention has become even more accentuated than before, as being one of the main problems on which hinges the success of armies in the field. Owing largely to the persistence with which Dr. Leigh Canney has hammered into the ears of more or less unwilling listeners the water aspect of the problem, something definite seems at last to have been done to ensure a more careful attention being given to supplying troops on the march with safe water. What has been done, however, and even what has been promised, falls far short of the logical suggestions made by Dr. Canney, and a lamentable torpor seems still to hang over the official mind in regard to this all-important question. The fact does not yet appear to be properly grasped that the provision of pure water is not a protection against enteric alone, but against a number of other debilitating and disabling diseases, and that the absence of enteric fever from an army in the field is not merely good in itself, but is good also as a proof that its sanitary wants are being well cared for. The matter, however, no longer lies with the medical profession; the doctors have pointed out the way, it is for the War Office to carry out the precepts. When we hear of a commanding officer finding himself seriously compromised at the War Office on account of the occurrence of a few unexplained cases of enteric in his regiment, then we shall have some hope for the British Army.

### Tuberculosis.

So far as the pathology and etiology of this disease are concerned, the interesting question raised by Professor Koch as to the non-identity of human and bovine tuberculosis remains practically where it was. Professor Koch has reiterated his opinion, and his opponents remain unconvinced. Meanwhile the matter is being investigated on behalf of the English and other Governments, and some time in the course of this year we may hope for a pronouncement on the subject. A matter of considerable interest in regard to sanatorium treatment has been the award by direction of the King of a large sum of money in prizes for the best essays upon "The Sanatorium Treatment of Consumptives," and not a little curiosity is likely to be evinced as to the results, when concrete form shall have been given to the products of this competition.

At the international conference on tuberculosis which was held in Berlin many old subjects and some new ones were discussed, and in the end a Central Bureau for the Prevention of Consumption was established, on the hypothesis, apparently, that combined international action is requisite for the eradication of the disease—all of which is an open question. It is true enough that peripatetic congresses—even international ones—do good by stirring up interest on the subject and inducing backward nations to emulate those which are in the van of sanitary progress; but when it is suggested that a permanent bureau of international action is required, except so far as food supply is concerned, in combating a disease which results from the prevalence of insanitary modes of life in each country and each district of each country we can but express our doubts. There is, moreover, a little touch of humour in the fact that Berlin should give birth to such a bureau, seeing that international action must chiefly have regard to the distribution of meat and milk infected with bovine tuberculosis, which the great Berlin professor holds to be something separate and apart from that human tuberculosis which it is the object of the bureau to combat.

### Cancer.

The mystery of cancer still remains unsolved; indeed so far as the etiology of the disease is concerned we do not find in the papers of the year anything which can be said to indicate solid advance, although several have appeared which were full of suggestiveness. In the treatment of the disease along the old lines of early operation and thorough removal there has, however, been progress on every side. By keeping strictly in view these two leading principles, by improved technique and more careful selection, the proportion of successes to operations appears to be rising. More especially, perhaps, is this the case in cancer of the uterus, the prospects of which disease when taken early enough are distinctly encouraging. Nor must one omit to record the growing favour into which the use of the X-rays is coming in the treatment of certain forms of malignant disease. In rodent ulcer this method has become fairly established; and although in other forms far less success has been obtained, one must remember that in nearly all the cases in which it has been employed the disease has been so far advanced as to have been already regarded as inoperable.

### The Vision of School Children.

At last, after long preaching on the subject, professional opinion as to the injury done to the rising generation by enforcing educational methods suitable to those with good sight upon children whose sight was imperfect has borne fruit, and the School Board of London have appointed a number of ophthalmic surgeons to visit their schools and test the sight of the children therein. Unfortunately this is but a step. To discover an abnormality or a disease is one thing, to treat it is quite another; and the result in London has been much the same as that recently reported from New York, where, on the same step being taken, a serious overcrowding of the out-patient departments of the eye hospitals has taken place. Directions are, we believe, given to the parents as to what course should be adopted in each case; but the London parent is a creature of habit, and so thoroughly engrained has become the habit of solving all medical questions by the simple recipe, "Take 'im to the 'orspital," that it becomes a question whether the School Board should not in equity be requested to pay something towards the extra work thus thrown upon these institutions.

### Appendicitis.

By the illness of the King attention was called in a striking and dramatic manner to the subject of appendicitis, and many disquisitions have been poured forth as to the treatment of this condition, which still remains the pivot around which revolve many academic discussions. The Americans still remain the principal advocates of immediate operation in all cases, but adherents of the same creed are not lacking here also, and Sir Frederick Treves and those associated with him in the treatment of the King in his illness had to run the gauntlet of much criticism in reference to the line they adopted in the case of his royal patient. There can be no doubt that the successful issue of this case and the many discussions to which it gave rise have had the effect of fixing more firmly than before in the mind of the profession the profound distinction which has to be drawn between acute and chronic appendicitis, and in emphasising the general principle that suppuration rather than appendicitis is the reason and excuse for operating in the acute stage, while the propriety of performing an "interval" operation does not depend upon the fact of the appendix having been inflamed, so much as upon the likelihood of such inflammation being repeated under the particular conditions of life to which the particular patient is likely to be exposed.

### Intra-peritoneal Hæmatocele.

Attention has been directed to the subject of ectopic gestation and the accidents which follow in its train by several papers and discussions, and in view of the constant and reiterated teaching with which for some years past medical literature has teemed, to the effect that intra-peritoneal hæmorrhage so caused should be treated by laparotomy with ligature or removal of the bleeding structure, we may almost take it as an advance, although one of reaction, to find Dr. Champneys asserting that of the cases suffering from this condition under his care at St. Bartholomew's Hospital, in no less than half recovery took place without operation. It was well that this should be stated, for although it was well known by those who have followed hospital practice that a good many such cases were being treated tentatively, the impression left by current ephemeral medical literature has certainly been in favour of a rush to operation.

### Surgery of the Stomach.

A great deal of attention has been given during the year to the surgery of the stomach; and although some extraordinary cases of extensive resection for cancerous growths have been recorded, it is evident that in regard to cancer, here as elsewhere, advance is being made rather in the direction of early and even exploratory operation rather than by way of more extensive operative procedures. Great advance has been made in converting the profession to the advantage and necessity of operation in cases of recurrent hæmatemesis; but a change is coming over the scene in regard to the operation to be performed, for it is being rendered evident that although excision of the ulcer and closure of the opening may be applicable in certain instances, there is a considerable range of diseases in the treatment of which gastro-enterostomy is the operation to be performed —a new example of the old principle of giving rest to a diseased organ. By short-circuiting the gastro-intestinal current, and so avoiding the obstructive action of the pylorus, rest is obtained.

### The Midwives Act.

Although somewhat outside the strict limits of medical work, normal midwifery must always be separated by such a thin line from that which is abnormal that the passing of the Midwives Act is a matter of much interest, especially to the rank and file of the profession. We can hardly avoid the fear that, at first, considerable friction and some irritation—the one indicative of the other—will be found to arise from this new departure, but should the Midwives Board be well advised, and should any real attempt be made to keep the midwives within their proper field of action, we cannot but feel that medical practitioners will in the end find their position much improved.

### Honours to Members of the Medical Profession.

First in rank and place, because most coveted even by the highest, must always stand that reward and recognition of personal bravery in the field, the Victoria Cross; and it is gratifying to record that this distinguished Order has been conferred upon two members of our profession during the year—Surgeon-Captain T. J. Crean, of the 1st Imperial Light Horse, and Surgeon-Captain A. Martin-Leake, of the South African Constabulary.

On the occasion of his coronation, his Majesty King Edward VII. instituted the new Order of Merit, and among the twelve eminent men chosen as the nucleus of this Order was the Right Hon. Lord Lister, who was at the same time made a member of the Privy Council. Baronetcies were on the same occasion conferred upon Sir Francis Laking, K.C.V.O., and Sir Frederick Treves, K.C.V.O., C.B.; and the following gentlemen were made knights: Dr. W. J. Collins, Mr. Alfred Cooper, Dr. J. Halliday Croom (President of the Royal College of Surgeons of Edinburgh), Dr. Thomas R. Fraser, F.R.S. (President of the Royal College of Physicians of Edinburgh), Mr. Victor Horsley, F.R.S., Mr. H. G. Howse (President of the Royal College of Surgeons of England), Mr. Henry Bell Longhurst, Professor William Macewen, F.R.S., Dr. Thomas Myles (President of the Royal College of Surgeons in Ireland), Dr. Isambard Owen, Professor William Whitla, Dr. Conan Doyle, and, let us add, as at one time an active member of our profession, Mr. Charles Wyndham.

The Hon. Dr. Frederick W. Borden, Minister of Militia and Defences of the Dominion of Canada, and Surgeon-General Pinching, head of the Sanitary Department at Cairo, were appointed Knights Commanders of the Order of St. Michael and St. George.

Inspector-General of Hospitals and Fleets John Denis Macdonald, R.N. (retired), Surgeon-General John Andrew Woolfryes, C.B, C.M.G., honorary physician to the King, Surgeon-General Annesley Charles Castriot de Renzy, C.B., I.M.S. (retired), and Surgeon-General (ranking as Lieutenant-General) William Taylor, C.B., honorary physician to the King, Director-General of the Army Medical Service, were each appointed to Knight Commanderships of the Bath.

### The Illustrious and the Forgotten Dead.

Death, as must every year be the case in regard to so hardworking a profession as that of medicine, has again been busy in our ranks, and has removed many a well-known figure from our midst—and, let us not forget, many a figure which although not well known had in his own more restricted field been held in honour, and many a one also who, although not much honoured in his own country, had at least done good work, and fought a good fight, and struggled to keep unsoiled the dignity of his profession. In death all men are equal; let us not make invidious selections. Let them rest in peace, and equally in the honour and respect of those who remain.

